# Detection of enterovirus in cerebrospinal fluids without pleocytosis in febrile infants under 3 months old reduces antibiotherapy duration

**DOI:** 10.3389/fped.2023.1122460

**Published:** 2023-02-28

**Authors:** Marion Blachez, Jeremy Boussier, Patricia Mariani, Caroline Caula, Jean Gaschignard, Alain Lefèvre-Utile

**Affiliations:** ^1^ General Pediatrics and Pediatric Emergency Department, Saint Camille Hospital, Bry-sur-Marne, France; ^2^ Sorbonne Université, La Pitié Salpêtrière Hospital, Paris, France; ^3^ Assistance Publique Hôpitaux de Paris (APHP), Laboratory of Microbiology, Robert Debré Hospital, Université de Paris, Paris, France; ^4^ Assistance Publique Hôpitaux de Paris (APHP), Pediatric Emergency Department, Robert Debré Hospital, Université de Paris, Paris, France; ^5^ General Pediatrics and Pediatric Emergency Department, Nord-Essonne Hospital Group, Longjumeau, France; ^6^ INSERM, UMR1137 - IAME, Université de Paris, Paris, France; ^7^ Assistance Publique-Hôpitaux de Paris (APHP), General Pediatric and Pediatric Emergency Department, Jean Verdier Hospital, Bondy, France; ^8^ INSERM U976 - Human Systems Immunology and Inflammatory Networks, Saint Louis Research Institute, Université de Paris, Paris, France

**Keywords:** enterovirus, meningitis, pleocytosis, antibiotherapy, infant

## Abstract

**Background:**

Infants under 3 months old with fever often receive empirical antibiotic treatment. *Enterovirus* is one of the leading causes of infection and aseptic meningitis but is not systematically screened. We aimed to evaluate enterovirus positive RT-PCR proportion in cerebrospinal fluid (CSF) with no pleocytosis and its impact on antibiotic treatment duration.

**Methods:**

During the enterovirus endemic season, from 2015 to 2018, we retrospectively studied infants under 3 months old, consulting for fever without cause, with normal CSF analysis, and receiving empirical antibiotic treatment. Clinical and biological data were analyzed, notably enterovirus RT-PCR results. The primary outcome was the duration of antibiotic therapy.

**Results:**

92 patients were recruited. When tested, 41% of infants were positive for enterovirus, median antibiotic duration was reduced in enterovirus positive in comparison to negative patients with respectively 1.9 [interquartile range (IQR), 1.7–2] vs. 4.1 [IQR, 2–6], *p* < 0.001. No clinical nor biological features differed according to the enterovirus status.

**Conclusion:**

In this population, enterovirus positive CSF are frequent despite the absence of pleocytosis. However, its research was not guided by clinical or biological presentations. Systematic and routine use of enterovirus RT-PCR during enterovirus season, regardless of CSF cell count, could reduce the prescription of antibiotics in febrile infants under 3 months old without clinical orientation.

## Introduction

Infants under 3 months old are at high risk of serious bacterial infection. Hence protocols have been established to manage fever in these infants, focusing on laboratory tests and antibiotic treatment. In this population, the etiology of fever is viral up to 67% (1) and bacterial in up to 12% (2) of cases. Fever remains often undocumented in about a third of these situations. Fever without source (i.e., without immediate clinical or microbiological orientation by rapid test as urine dipstick or antigenic viral test, FWS) is a frequent cause of consultation in the emergency department (ED) and a close follow-up is recommended to limit antibiotic use for patients at low risk of serious bacterial infection (3). Occult bacteremia only accounts for 2% of infants under 3 months old with FWS (4).

*Enterovirus* infection is a common cause of fever and the most frequent etiology of aseptic meningitis (5). The detection of enterovirus from cerebrospinal fluid (CSF) by reverse-transcription polymerase chain reaction (RT-PCR) has been recommended in the diagnosis of meningitis (6). Routine enterovirus testing by RT-PCR in CSF of febrile infants under 3 months old could reduce the length of hospitalization (7). However, there is no consensus on the indications for performing enterovirus RT-PCR which may differ from one hospital to another (8). Additionally, the absence of pleocytosis in CSF may limit the prescription of an enterovirus RT-PCR.

The main objective of our study was to assess the proportion of enterovirus RT-PCR positive testing in CSF with no pleocytosis and its impact on antibiotherapy duration, in infants under 3 months old treated by antibiotics for FWS. The secondary objective was to determine whether clinical or biological features could characterize patients with different enterovirus RT-PCR statuses.

## Methods

We performed a retrospective study in the ED of Robert Debré University Hospital, Paris, France, during the 4 consecutive endemic seasons of enterovirus (April to September) between 2015 and 2018, according to Santé Publique France observation (9). We included infants under 3 months old presenting with FWS who had a lumbar puncture with no pleocytosis and were initially treated with antibiotics. FWS was defined by the absence of an immediate clinical or microbiological orientation by rapid test as urine dipstick or antigenic viral test, no evidence of respiratory or cutaneous infection, and the absence of bacteria on direct examination of urine or CSF. Enterovirus was screened in CSF using the GeneXpert enterovirus RT-PCR assay (GXEA; Cepheid, Sunnyvale, CA).

Clinical, biological, and microbiological data were extracted from electronic medical records, including risk factors of neonatal bacterial infection, as defined in French guidelines (10). Respiratory signs were defined by the presence of dyspnea, apnea, or hypoxia. Hemodynamic signs included the need for volume expansion, tachycardia, or signs of peripheral hypoperfusion. Neurologic signs were defined as the presence of asthenia, hypotonia, irritability, consciousness alteration, or seizure. Gastrointestinal symptoms included vomiting, diarrhea, and feeding difficulties. If a clinical sign was not mentioned in a record, it was considered absent. Biological features recorded were C-reactive protein (CRP), procalcitonin (PCT), and white blood cell count. Pleocytosis was defined as ≥15 cells in the CSF for infants under 28 days of age, and ≥10 for infants aged 29–90 days (11). For hemorrhagic CSFs (>1,000 red blood cells/mm^3^), we applied the 1:1,000 white blood cell correction formula detailed in Lyons et al. (12).

Statistics were performed and graphical representations were created using R (CRAN) version 3.6.0. Categorical variables were expressed as absolute numbers and percentages and compared using the chi-squared test or Fisher’s exact test, while continuous data were described as median with interquartile and compared the Kruskal–Wallis test. The log-rank test was used to compare the duration of antibiotic therapy, either between all three groups, or in one-by-one comparisons. Sensitivity analysis for the log-rank test was performed in infants under 2 months old. We performed a linear regression with adjustment for age, log(CRP), log(PCT), and the presence of at least one hemodynamic or neurological sign to evaluate how enterovirus RT-PCR status could modify antibiotic treatment duration independently to these factors. Linear regression assumptions were assessed either graphically or numerically. There were no influential outliers. Two-sided *p*-values <0.05 were considered significant. No correction for multiple testing was performed.

The study was approved by the ethics committee of the hospital and has been declared to the CNIL (French data protection authority) under the number 2208734v0.

## Results

**Figure 1:**
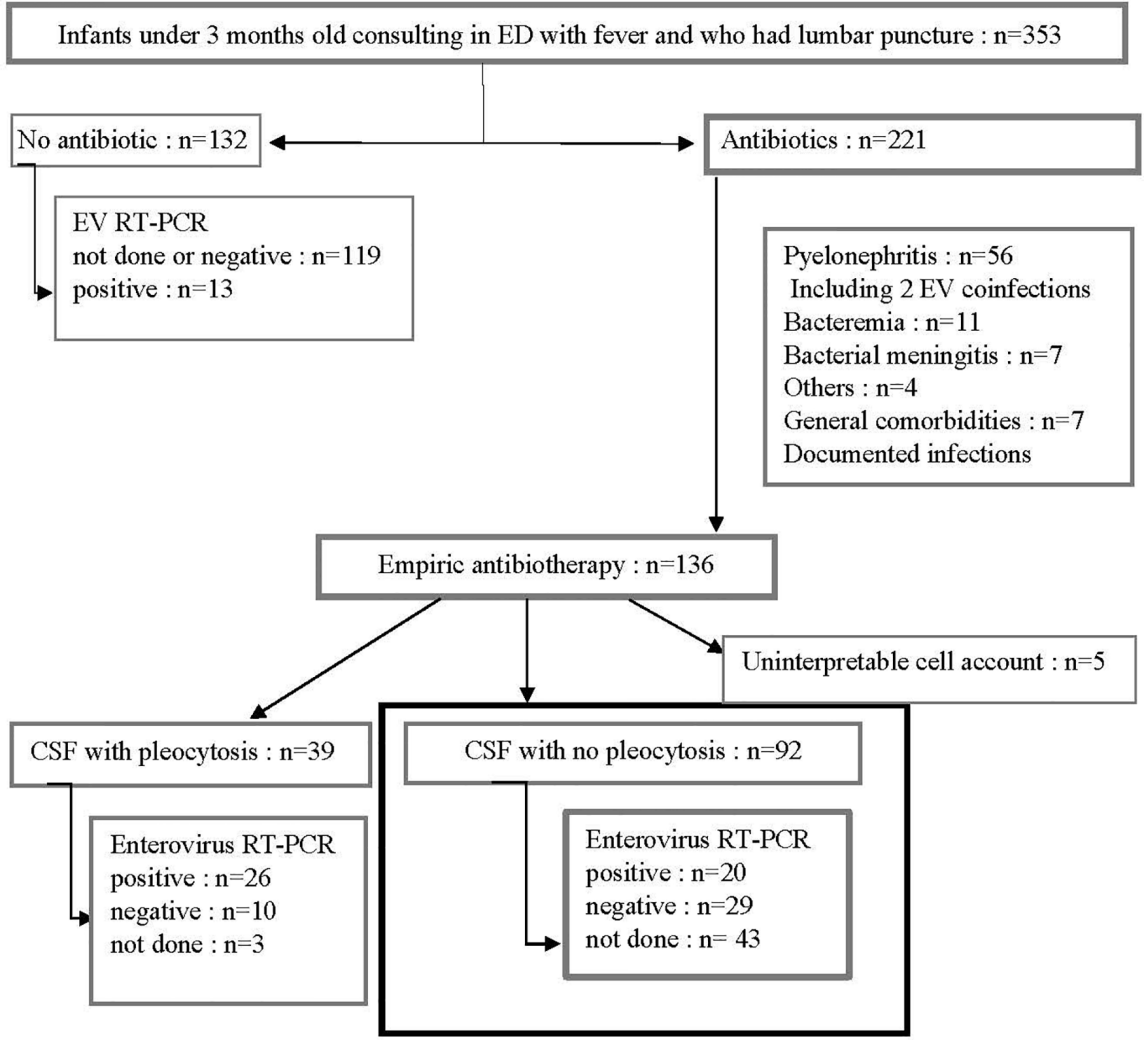
Patient flow chart. During four consecutive EV endemic periods, all febrile infants under 3 months old who had a lumbar puncture performed were screened. We selected those who received empiric antibiotic treatment, with no apparent cause of fever, and without pleocytosis (bold square). ED, emergency department; EV, enterovirus; RT-PCR, reverse transcriptase polymerase chain reaction.

During the study period, 353 infants under 3 months old admitted to the ED had a lumbar puncture performed, of which 221 received antibiotics, of which 136 were diagnosed as FWS. A total of 92/136 (67%) infants had a CSF with no pleocytosis and constituted our study population. Patients excluded for documented infections included cases of pyelonephritis (*n* = 56, two of whom had enterovirus coinfections), bacteremia (*n* = 11), bacterial meningitis (*n* = 7) (Figure 1). CSF cell count could not be interpreted for 5 infants. Baseline characteristics of these 92 infants are shown in Table 1. The age of patients was similar between the three groups: *unperformed-enterovirus* RT-PCR, *enterovirus-positive*, and *enterovirus-negative* infants with respectively a median of 27, 23, and 26 days (*p* = 0.53). No significant difference in terms of neonatal history, clinical features, blood, or CSF analysis was found between the three groups. In particular, groups did not differ for clinical signs of sepsis with respectively 36%, 25%, 29% (*p* = 0.65) of hemodynamic signs for *unperformed*, *positive*, and *negative* infants. Median CRP was respectively 12, 17, and 10 mg/L in each group (*p* = 0.64) and median PCT was similar with respectively 0.2, 0.2, and 0.3 µg/L (*p* = 0.90). Median white blood cell counts were respectively 8.1, 8.6, and 8.8 G/L for each group, with similar proportions of polymorphonuclear neutrophils (PMN) and lymphocytes. An enterovirus RT-PCR was performed in the CSF of 49/92 (53%) infants. It was positive in 20 (i.e., 41% of tested and 22% of all infants), while negative in 29 (i.e., 59% of tested infants).

**Table 1:** Clinical and biological features according to EV RT-PCR status in children without pleocytosis.

Total patients (*n* = 92)	Not tested for EV (*N* = 43)	EV RT-PCR (+) (*N* = 20)	EV RT-PCR (−) (*N* = 29)	*p*
Age (days)	27 [IQR, 4–85]	23 [IQR, 5–64]	26 [IQR, 6–53]	0.53
Neonatal history				
Preterm birth	1 (2)	2 (10)	2 (7)	0.39
Risk factor of bacterial infection	12 (28)	8 (40)	9 (31)	0.97
Clinical features				
Fever 38°C–39°C	35 (81)	14 (70)	22 (76)	0.59
Fever >39°C	6 (14)	6 (30)	6 (21)	0.32
Respiratory signs	5 (12)	2 (10)	6 (21)	0.44
Hemodynamic signs	15 (36)	5 (25)	8 (29)	0.65
Neurologic signs	21 (50)	12 (60)	21 (75)	0.11
Gastrointestinal signs	20 (47)	6 (30)	13 (45)	0.44
CSF analysis				
White blood cells (/mm^3^)	3 [IQR, 0–11]	1 [IQR, 0–8]	2 [IQR, 0–13]	-
Red blood cells >1,000/mm^3^	11 (26)	10 (50)	9 (31)	0.17
Proteins (g/L)	0.43 [IQR, 0.26–4.8]	0.71 [IQR, 0.22–6.76]	0.52 [IQR, 0.24–0.98]	0.13
Glucose (mmol/L)	3.2 [IQR, 2.3–5.1]	3.15 [IQR, 2.3–3.8]	3.2 [IQR, 1.7–6.7]	0.44
Blood analysis				
CRP (mg/L)	12 [IQR, 0.1–131]	17 [IQR, 0.1–138]	10 [IQR, 0.1–199]	0.64
PCT (µg/L)	0.2 [IQR, 0.1–26]	0.2 [IQR, 0.1–2.1]	0.3 [IQR, 0.1–32]	0.90
White blood cells (G/L)	8.1 [IQR, 4.0–29.2]	8.6 [IQR, 4.3–14.7]	8.8 [IQR, 3.3–33.1]	0.83
PMN (G/L)	4.0 [IQR, 0.7–23.7]	5.2 [IQR, 2.2–32.5]	4.7 [IQR, 0.9–21.6]	0.20
Lymphocytes (G/L)	2.8 [IQR, 1.0–11.5]	3.1 [IQR, 1.3–6.7]	1.8 [IQR, 0.6–6.2]	0.34

Kruskal–Wallis test was used to compare continous variables, and *χ*^2^ test of independence or Fisher’s exact test was used for categorical variables. CRP, C-reactive protein; EV, enterovirus; PCT, procalcitonin; PMN, polymorphonuclear neutrophils; RT-PCR, reverse transcriptase polymerase chain reaction; IQR, interquartile range. Absolute values and percentage are provided for categorical variables. Median, minimum and maximum are provided for quantitative variables.

**Figure 2:**
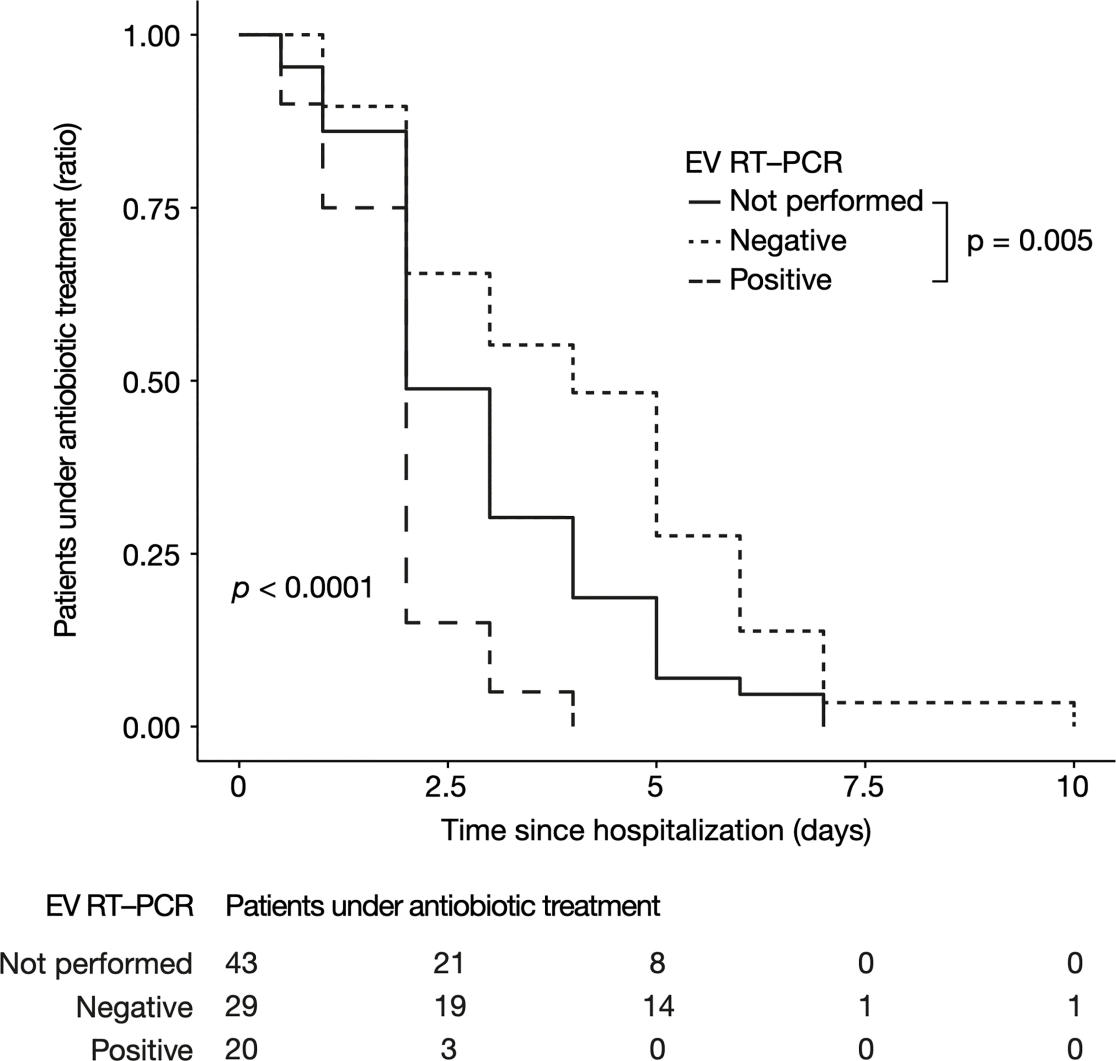
Antibiotic treatment duration according to EV RT-PCR status in children without pleocytosis. Represented as a Kaplan-Meier curve. The *p*-value was calculated by the log-rank test. EV, enterovirus; RT-PCR, reverse transcriptase polymerase chain reaction.

Antibiotic treatment duration was significantly shorter for *enterovirus-positive* CSF patients, with a median of 1.9 [interquartile range (IQR), 1.7–2], 4.1 [IQR, 2–6], and 2.2 [IQR, 1–4], days for *positive*, *negative*, and *unperformed* group respectively (*p* < 0.001) (Figure 2). In the linear regression which evaluates the association between enterovirus RT-PCR result and treatment duration, a positive enterovirus RT-PCR reduced the duration of antibiotic treatment by 1.03 day (95% confidence interval [IQR, −1.96, −0.1], *p* = 0.03) compared to patients for which enterovirus RT-PCR was not performed, while a negative enterovirus RT-PCR increased it by 1.17 day (95% CI [IQR, 0.35, 2], *p* = 0.005). Linear regression with adjustment for age, CRP, PCT, and the presence of hemodynamic or neurological signs yielded similar results: a decrease in antibiotic treatment by 1.28 day (95% CI [IQR, −2.18, −0.39], *p* = 0.005) for positive enterovirus RT-PCR compared to patients for which enterovirus RT-PCR was not performed, and an increase by 1.04 day (95% CI [IQR, 0.28, 1.81], *p* = 0.008) for negative enterovirus RT-PCR. Hospitalization duration was significantly reduced for positive enterovirus RT-PCR infants, in multivariate analysis (result available upon request). Together, these data suggest an impact of enterovirus RT-PCR results on antibiotic treatment duration, independent of other clinical and biological features.

## Discussion

Our study was carried out in a large French pediatric ED during four consecutive enterovirus endemic seasons. It documents the proportion and description of infants presenting with fever without source associated with CSF-positive for enterovirus in the absence of pleocytosis, and studies its impact on antibiotic treatment duration.

We found that during the endemic period, enterovirus could be detected in 22%–41% of infants with an apparently normal CSF. Among patients treated empirically with antibiotics (*n* = 136), enterovirus RT-PCR was performed in 88 of them. There was no significant difference between their initial clinical presentations. The significant parameters between the two groups were the number of elements in the CSF and the protein count, suggesting that the presence of pleocytosis leads the clinician to request an enterovirus RT-PCR (Results available on Appendix S1). Additionally, proven enterovirus infection led to a decreased antibiotic treatment duration of 1.28 day in comparison to non-tested infants. Moreover, without pleocytosis, neither symptoms, CSF parameters nor inflammation seemed to indicate whether an enterovirus RT-PCR would be prescribed or predict its result.

Infants with a proven uncomplicated enterovirus infection benefit from a shorter treatment and hospitalization period (13). Yet our study is the first one to report the impact on enterovirus screening in apparently normal CSF, with a significant decrease in antibiotic duration when detected. We consider that a 1-day-difference in antibiotic duration is clinically significant for patients and families, as it is also correlated with a shortening of hospitalization duration. While enterovirus RT-PCR positivity results in a shorter treatment duration, we noticed that when RT-PCR is negative, the duration of antibiotic therapy is increased. This result contradicts the recent finding of Paioni et al. in which treatment duration is comparable when enterovirus RT-PCR is negative or not done. But the latter did not focus on CSF without pleocytosis (13). This paradox may reflect the cautious approach of clinicians treating culture-negative sepsis.

Among the 136 children treated by antibiotics, 112 had a dual therapy using aminoside and third generation cephalosporin. Seven children had 3 antibiotics. Meningeal dose was directly prescribed for 90 patients. Thus, we hypothesize that enterovirus RT-PCR results could assist the clinician and promote the standardization of practices.

Enterovirus CSF infections without meningitis have often been reported in large cohort studies about enterovirus infections, but no study focused on this situation in infants. In this work, the absence of pleocytosis was found in 44% of enterovirus CSF infections, a finding which is consistent with the 16%–68% observed in the literature (14, 15). In a Japanese cohort of 263 febrile infants under 4 months old, the use of enterovirus RT-PCR enabled the diagnosis of 60% of aseptic meningitis and 33% of FWS (16). In addition, up to 83% of under 3 months old with *enterovirus-positive* CSF presented no pleocytosis (17). Some authors have suggested that normal CSF was related to younger age (under 2 months old) and other independent factors such as peripheral white blood cell count and time of lumbar puncture (14, 15, 18, 19). This is supported by the fact that the instruction given to parents is to consult early in case of fever of an infant under 3 months old. At this early time point, it is possible that the inflammatory response has not begun, while the virus is already present and can be detected.

Notably, clinical and biological characteristics did not significantly differ between groups, suggesting that no particular sign can help clinicians to predict the usefulness or the result of enterovirus RT-PCR. This finding is in line with the results of Eichinger et al., who observed no differences in terms of symptoms and laboratory parameters in patients with or without viral meningitis (20).

A positive enterovirus RT-PCR cannot completely rule out bacterial coinfection. In excluded patients, we found two *enterovirus-positive* RT-PCR among infants with a diagnosis of urinary tract infections. This might be expected since diagnosis of urinary tract infection may be overreported because of the risk of contamination during urine collection. Nigrovic et al. studied a large population of 3,295 infants with pleocytosis and found no bacterial coinfection in CSF associated with enterovirus detection (21). However, clinicians must remain highly vigilant, especially if the lumbar puncture has occurred after the administration of antibiotics (22).

Our study has several limitations, inherent to its monocentric and retrospective design which can limit its external validation. This is why we have reduced the number of missing data by focusing on a limited number of variables. However, several reasons make us believe that our population is probably close to the general population of infants under 3 months old admitted to the ED. The rate of the different types of infection (bacterial, viral…) found in our population was similar to other studies (23).

Even when febrile infants were tested for enterovirus, 59% of them didn’t have any proven infection in the end. In this population, antibiotic therapy duration was significantly longer. We believe that they could benefit from a broader and a more systematic CSF microbiological screening. We propose that enterovirus RT-PCR and possibly CSF multiplexed PCR (screening up to several dozen microbes) should be prescribed more routinely to infants treated with antibiotics, to detect potential unexpected viral infections, limit the duration of treatment, thereby avoiding adverse effects, and generating potential public health savings. This strategy showed positive results in pediatric departments that implemented systematic CSF screening in children with FWS (24, 25). The cost of a multiplex PCR panel is higher than the GeneXpert (177 USD vs. 35 USD per test) but it has the benefit of detecting several pathogens with short delivery results.

## Conclusion

Infants under 3 months old presenting with fever without a source are often treated with empiric antibiotic treatment, while they could be infected with enterovirus. In our cohort, enterovirus was detected in 41% of tested CSF without pleocytosis. Its detection helped to understand the microbiological diagnosis of febrile infants and led to a significant reduction of treatment duration. Systematic and routine use of enterovirus RT-PCR, regardless of CSF cell count, could reduce the prescription of antibiotics in febrile infants under 3 months old with no clinical orientation.

## Data Availability

The original contributions presented in the study are included in the article/**Supplementary Material**, further inquiries can be directed to the corresponding author.

## References

[1] Burstein B Anderson G Yannopoulos A. Prevalence of serious bacterial infections among febrile infants 90 days or younger in a Canadian urban pediatric emergency department during the COVID-19 pandemic. JAMA Netw Open. (2021) 4:e2116919. 10.1001/jamanetworkopen.2021.16919 34255052 PMC8278260

[2] Chen Y-T Chang Y-J Liu B-Y Lee E-P Wu H-P. Severe bacterial infection in young infants with pyrexia admitted to the emergency department. Medicine. (2021) 100:e26596. 10.1097/MD.0000000000026596 34232210 PMC8270585

[3] Hernandez-Bou S Trenchs V Batlle A Gene A Luaces C. Occult bacteraemia is uncommon in febrile infants who appear well, and close clinical follow-up is more appropriate than blood tests. Acta Paediatr Oslo Nor. (2015) 104:e76–81. 10.1111/apa.12852 25378087

[4] Bressan S Berlese P Mion T Masiero S Cavallaro A Dalt LD. Bacteremia in feverish children presenting to the emergency department: a retrospective study and literature review. Acta Paediatr. (2012) 101:271–7. 10.1111/j.1651-2227.2011.02478.x 21950707

[5] Rotbart HA McCracken GH Jr Whitley RJ Modlin JF Cascino M Shah S Clinical significance of enteroviruses in serious summer febrile illnesses of children. Pediatr Infect Dis J. (1999) 18:869–74. 10.1097/00006454-199910000-00007 10530582

[6] Archimbaud C Ouchchane L Mirand A Chambon M Demeocq F Labbé A Improvement of the management of infants, children and adults with a molecular diagnosis of enterovirus meningitis during two observational study periods. PLoS One. (2013) 8:e68571. 10.1371/journal.pone.0068571 23874676 PMC3708915

[7] Wallace SS Lopez MA Caviness AC. Impact of enterovirus testing on resource use in febrile young infants: a systematic review. Hosp Pediatr. (2017) 7:96–102. 10.1542/hpeds.2016-0060 28082417

[8] Baker MD Avner JR. Management of fever in young infants: evidence versus common practice. Pediatrics. (2016) 138:e20162085. 10.1542/peds.2016-2085 27940707

[9] Santé Publique France. Point sur les infections à entérovirus au 7 août 2013. Available at: https://www.santepubliquefrance.fr/maladies-et-traumatismes/maladies-a-prevention-vaccinale/poliomyelite/documents/bulletin-national/point-sur-les-infections-a-enterovirus-au-7-aout-2013.

[10] Société française de néonatologie et Société française de pédiatrie. Prise en charge du nouveau-né à risque d’infection néonatale bactérienne précoce (≥ 34 SA). Perfect En Pédiatrie. (2018) 1:10–8. 10.1016/j.perped.2018.01.011

[11] Thomson J Sucharew H Cruz AT Nigrovic LE Freedman SB Garro AC Cerebrospinal fluid reference values for young infants undergoing lumbar puncture. Pediatrics. (2018) 141(3):e20173405. 10.1542/peds.2017-3405 29437883

[12] Lyons TW Cruz AT Freedman SB Neuman MI Balamuth F Mistry RD Interpretation of cerebrospinal fluid white blood cell counts in young infants with a traumatic lumbar puncture. Ann Emerg Med. (2017) 69:622–31. 10.1016/j.annemergmed.2016.10.008 28041826 PMC5406248

[13] Paioni P Barbey F Relly C Meyer Sauteur P Berger C. Impact of rapid enterovirus polymerase chain reaction testing on management of febrile young infants <90 days of age with aseptic meningitis. BMC Pediatr. (2020) 20:166. 10.1186/s12887-020-02066-0 32299396 PMC7161008

[14] Yun KW Choi EH Cheon DS Lee J Choi CW Hwang H Enteroviral meningitis without pleocytosis in children. Arch Dis Child. (2012) 97:874–8. 10.1136/archdischild-2012-301884 22814522

[15] Lumley SF Pritchard D Dutta A Matthews PC Cann K. Multiplex PCR reveals high prevalence of enterovirus and HHV6 in acellular paediatric cerebrospinal fluid samples. J Infect. (2018) 77:249–57. 10.1016/j.jinf.2018.05.008 29852190

[16] Kawashima H Kobayashi K Aritaki K Takami T Ioi H Kashiwagi Y Diagnosis and evaluation of febrile infants under 4 months of age in Japan by using RT-PCR for enterovirus. J Infect. (2006) 53:16–20. 10.1016/j.jinf.2005.09.017 16309745

[17] Ko Y Jeon W Chae MK Yang H Lee J. Clinical characteristics of enteroviral meningitis without pleocytosis in children: a retrospective single center observational study in the Republic of Korea. BMC Pediatr. (2019) 19:335. 10.1186/s12887-019-1714-1 31521164 PMC6744706

[18] Mulford WS Buller RS Arens MQ Storch GA. Correlation of cerebrospinal fluid (CSF) cell counts and elevated CSF protein levels with enterovirus reverse transcription-PCR results in pediatric and adult patients. J Clin Microbiol. (2004) 42:4199–203. 10.1128/JCM.42.9.4199-4203.2004 15365011 PMC516328

[19] de Crom SCM van Furth MAM Peeters MF Rossen JWA Obihara CC. Characteristics of pediatric patients with enterovirus meningitis and no cerebral fluid pleocytosis. Eur J Pediatr. (2012) 171:795–800. 10.1007/s00431-011-1626-z 22102153

[20] Eichinger A Hagen A Meyer-Bühn M Huebner J. Clinical benefits of introducing real-time multiplex PCR for cerebrospinal fluid as routine diagnostic at a tertiary care pediatric center. Infection. (2019) 47:51–8. 10.1007/s15010-018-1212-7 30187216

[21] Nigrovic LE Kuppermann N Macias CG Cannavino CR Moro-Sutherland DM Schremmer RD Clinical prediction rule for identifying children with cerebrospinal fluid pleocytosis at very low risk of bacterial meningitis. J Am Med Assoc. (2007) 297:52–60. 10.1001/jama.297.1.52 17200475

[22] Basmaci R Mariani P Delacroix G Azib S Faye A Muhamed-Kheir T Enteroviral meningitis does not exclude concurrent bacterial meningitis. J Clin Microbiol. (2011) 49:3442–3. 10.1128/JCM.01015-11 21878585 PMC3165612

[23] Milcent K Gajdos V. Use of procalcitonin assays to predict serious bacterial infection in young febrile infants-reply. JAMA Pediatr. (2016) 170:623–4. 10.1001/jamapediatrics.2016.0385 27088558

[24] Chakrabarti P Warren C Vincent L Kumar Y. Outcome of routine cerebrospinal fluid screening for enterovirus and human parechovirus infection among infants with sepsis-like illness or meningitis in Cornwall, UK. Eur J Pediatr. (2018) 177:1523–9. 10.1007/s00431-018-3209-8 30022279

[25] King RL Lorch SA Cohen DM Hodinka RL Cohn KA Shah SS. Routine cerebrospinal fluid enterovirus polymerase chain reaction testing reduces hospitalization and antibiotic use for infants 90 days of age or younger. Pediatrics. (2007) 120:489–96. 10.1542/peds.2007-0252 17766520

