# Supplementary figures and images for: Detection of enterovirus in cerebrospinal fluids without pleocytosis in febrile infants under 3 months old reduces antibiotherapy duration
Source: Front Pediatr. 2023 Feb 28;11:1122460. doi: 10.3389/fped.2023.1122460 (PMC10011150; doi:10.3389/fped.2023.1122460)

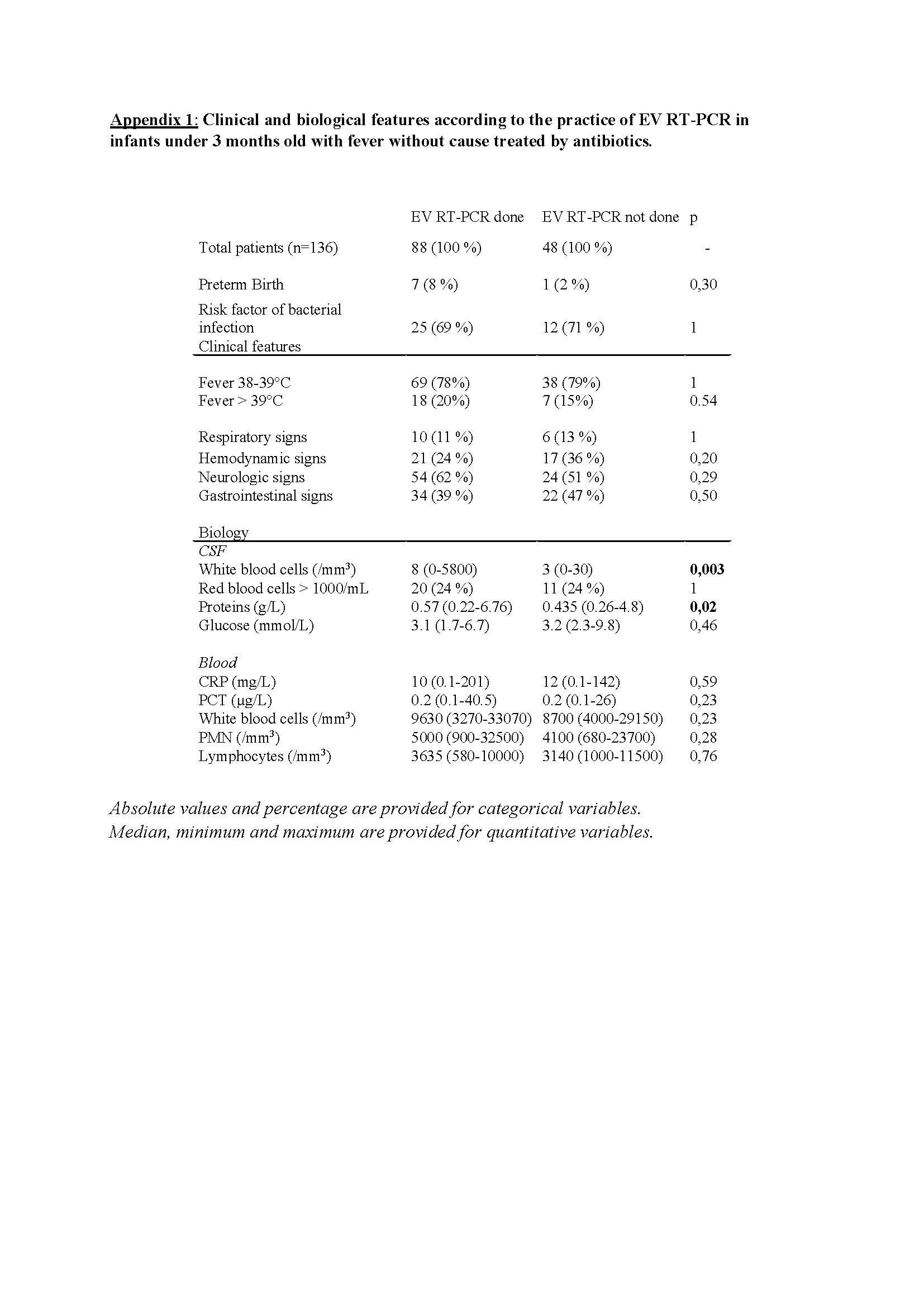

Supplement: Supplementary file 1 [file Image1.jpeg]

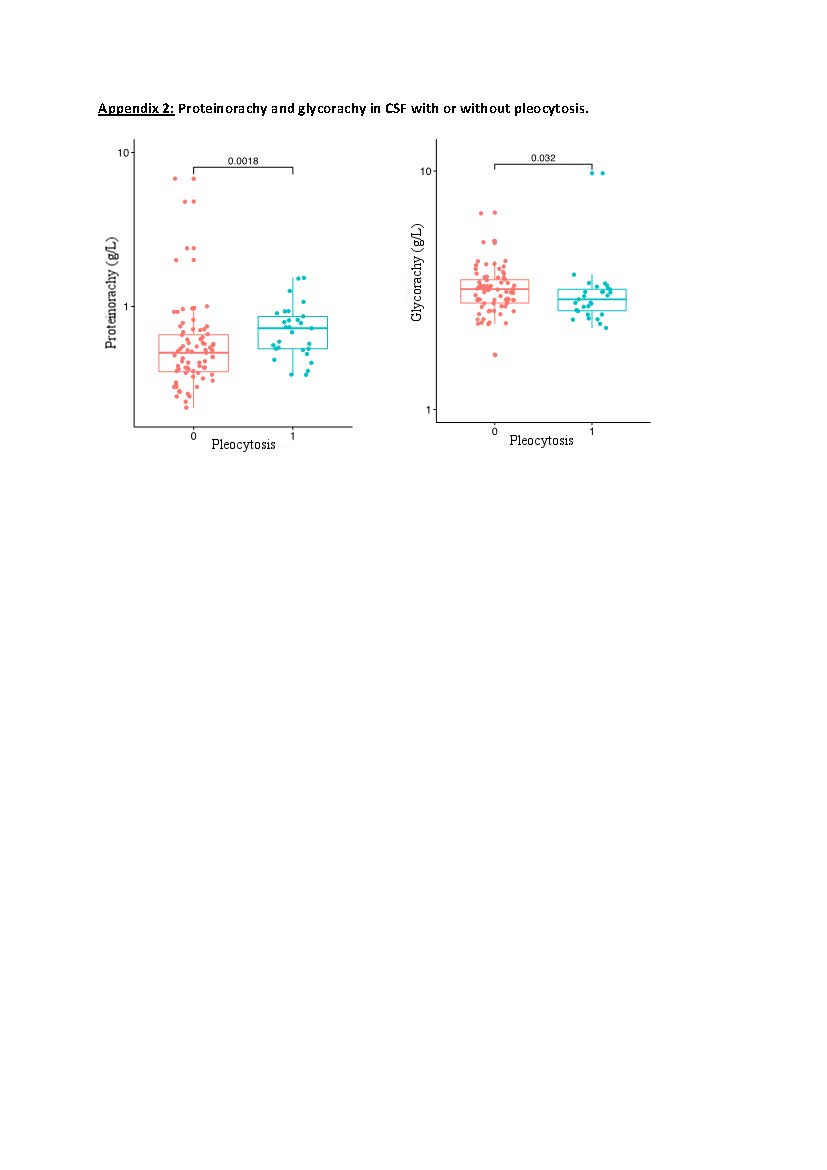

Supplement: Supplementary file 2 [file Image2.jpeg]
